# Unravelling the relation between altruistic cooperativeness trait, smiles, and cooperation: a mediation analysis

**DOI:** 10.3389/fpsyg.2023.1227266

**Published:** 2023-08-22

**Authors:** Xiaoqi Deng, Sarinasadat Hosseini, Yoshihiro Miyake, Takayuki Nozawa

**Affiliations:** ^1^Department of Computer Science, Tokyo Institute of Technology, Meguro City, Tokyo, Japan; ^2^Department of Intellectual Information Engineering, University of Toyama, Toyama, Japan

**Keywords:** smile, cooperation, personality, non-verbal behavior, synchrony

## Abstract

**Introduction:**

Human cooperativeness is an important personality trait. However, the mechanism through which people cooperate remains unclear. Previous research suggests that one of the proposed functions of smiling is to advertise altruistic dispositions, leading to successful cooperation. In particular, studies have reported that Duchenne smiles are honest signals of cooperative intent because they are not easy to produce voluntarily. This study aimed to examine the predictive relationships among altruistic cooperativeness traits, Duchenne smiles, and cooperative behavior.

**Methods:**

A total of 90 people were randomly assigned to dyads and filmed while they participated in a ten-minute, unstructured conversation followed by a prisoner’s dilemma game to measure their cooperative behaviors. Their smiles during conversations were classified as Duchenne or non-Duchenne. Participants’ altruistic dispositions were measured before the conversation began using an anonymous prisoner’s dilemma game.

**Results:**

The results of our linear regression analyses support previous findings that individual’s Duchenne smiles and their own cooperative behavior are positively correlated. However, when we controlled for altruistic cooperativeness, Duchenne smiles no longer correlated with cooperative behavior. The results of the mediation analyses showed that Duchenne smiles and smile synchrony did not mediate the predictive relationship between altruistic cooperativeness and cooperative behavior.

**Discussion:**

Our results suggest that human cooperative behavior may be predetermined by altruistic cooperativeness. This calls for the reconsideration of the Duchenne smile as an underlying behavioral mechanism that is effective for signaling altruistic cooperative intent.

## 1. Introduction

Cooperation is one of the most central aspects of human social behavior. Numerous experimental studies have demonstrated that in many situations, people choose to cooperate with non-kin individuals instead of pursuing their own short-term interest in anonymous interactions (Keser and Van Winden, 2000; Henrich, 2004; Cadsby et al., 2007; Barragan-Jason et al., 2021). Why and how people cooperate in a competitive world has puzzled researchers in evolutionary biology, psychology, and economics.

From the point of view of natural selection, animals (including human beings) pursuing their own self-interests rather than cooperation have superior survival rate because selfish behaviors are more competitive. However, in many situations, cooperation can lead to substantial gains for both the group that achieves cooperation and its individual members (Nowak, 2006; West et al., 2007). In some situations, individuals face the choice of either pursuing their own self-interests or cooperating with others to maximize collective interests. Should they choose the latter, they are at risk of exposing themselves to possible exploitation by “cheaters.” Therefore, the ability to detect cooperative partners is important to individuals. Human cooperativeness has been construed as a personality trait (Sommet et al., 2022). Cooperativeness can be measured using self-reported scales (Cloninger et al., 1993; Lu et al., 2013), as well as behavior measures (e.g., the prisoner’s dilemma game). Explaining how individuals identify cooperative partners and decide whether to cooperate remains a central focus in the study of human behavior.

The theory of reciprocal altruism (Trivers, 1971) has been widely accepted by many researchers who attempt to explain cooperation. Reciprocal altruism describes the social interactions between unrelated individuals with mutual benefits when they engage in reciprocal exchanges while attempting to avoid being cheated. Trivers (1971) suggested that humans have evolved to possess many psychological characteristics that enable them to detect cheaters and maintain the functioning of reciprocal altruism, and that emotions of liking play an important role. Recent research suggests that several underlying behavioral mechanisms may be responsible for the maintenance of strategies based on reciprocal altruism (Brown et al., 2003; Mehu et al., 2007a).

A rich body of research suggests that the key to successful cooperation is the ability to signal and detect the intention to cooperate via non-verbal behavior in order to identify cooperative partners. Many studies examining the functional use of facial expressions in cooperation during social interactions suggest that smiling could be a reliable signal of cooperative intentions. Reed et al. (2012) investigated the role of facial expressions in prisoner’s dilemma games and reported that smiles are predictive of cooperative decisions in dyads. Additionally, empirical research suggests that smiling is related to both the sender’s cooperative intent and the receiver’s level of trust in the sender. For example, cooperative and altruistic individuals display higher levels of smiles than do their non-cooperative counterparts (Brown et al., 2003; Mehu et al., 2007a). Other studies have found that individuals displaying enjoyment smiles tend to be rated as more trustworthy and selected for cooperative activities (Johnston et al., 2010), and that people are more cooperative with strangers represented by a smiling photograph in a trust game (Scharlemann et al., 2001). However, smiles can easily be faked (Gunnery et al., 2013). Okubo et al. (2012) reported that cheaters use fake smiles on the left side of their faces to conceal their uncooperative attitudes. Since people may smile to give an impression of cooperativeness in resource exploitation, the topic of why smiles continue to be used to guide cooperation has been attracting significant interest. This puzzle has been extensively discussed in the literature regarding animal signals. Many researchers have proposed that imposing a cost on the sender can maintain a signal’s honesty (Scott-Phillips, 2008; McCullough and Reed, 2016). Thus, by imposing costs on smiles, smiling can become a costly signal.

The existing literature on smiling suggests that Duchenne smiles (characterized by the use of the orbicularis oculi and the zygomatic major, which present the rise of the corners of the mouth as well as the wrinkles around the eyes) are cognitively costly to the smiler (Centorrino et al., 2015) and could be considered a reliable indicator of cooperative intent because only a few individuals are able to voluntarily control them (Ekman and Friesen, 1982; Schmidt and Cohn, 2001). Furthermore, several studies have demonstrated the relationship between Duchenne smiles and cooperative intent. There is evidence that senders expressing smiles are more likely to cooperate, and this effect is stronger for Duchenne smiles than for non-Duchenne smiles (Reed et al., 2012). Mehu et al. (2007b) put forward that a Duchenne smile could be an important signal for the maintenance of cooperative relationships. Pentland (2010) proposed that honest signals in human communication may take the form of mimicry or synchrony. Danvers and Shiota (2018) testified to the theoretical argument that dynamically engaged social displays can be considered honest signals, and their finding that when an interaction partner smiles in a responsive, dynamically engaged manner, the actor becomes more likely to cooperate partially supports this argument.

Some studies suggest that a Duchenne smile can reliably advertise altruistic intentions; thus, smiles can reliably signal cooperation (Mehu et al., 2007a; Oda et al., 2009). This idea indicates that smiles may mediate the effect of altruism on cooperative interaction. However, other studies have reported that smiles may also elicit cooperation from others (Guéguen and De Gail, 2003; Johnston et al., 2010). However, despite these findings, the predictive relationship between smiling and cooperation remains vague; that is, whether people with the characteristic of cooperativeness are more likely to smile or cooperate. Furthermore, although there is plentiful evidence that altruism can be favored when recipients are relatives or reciprocators, a growing body of literature shows that altruism cannot explain many cooperative behaviors (Fehr and Fischbacher, 2003; Roberts, 2005; Piff et al., 2010).

In the current study, we conducted a mediation analysis to test a hypothetical predictive chain in which the effect of an individual’s altruistic cooperativeness trait on successive cooperative behavior is mediated or explained by smiling, which is assumed to advertise cooperative intent. A total of 90 people were randomly assigned to dyads and filmed while they participated in a 10-min, unstructured conversation followed by a prisoner’s dilemma game to measure their cooperative behaviors. Their smiles during conversations were classified as Duchenne or non-Duchenne. The distinguishing feature of the current study is that before the conversation, the individual participants’ altruistic cooperativeness traits were measured using a one-shot, anonymous prisoner’s dilemma game with an experimenter. One advantage of using this method to measure altruistic cooperativeness is that it avoids the problem of social desirability bias in self-reported cooperativeness. Given previous findings, we are particularly interested in Duchenne smiles and Duchenne smile synchrony, which are assumed to be reliable signals of cooperation. First of all, we hypothesized that human cooperative behavior is influenced by individuals’ altruistic cooperativeness traits. Additionally, we hypothesized that Duchenne smiles and Duchenne smile synchrony are predicted by individuals’ altruistic cooperativeness traits, and, in turn, predict their own successive cooperative behavior.

## 2. Materials and methods

### 2.1. Participants

Ninety university and college students (42 female and 48 male) in Tokyo, Japan were recruited for this study. All participants had normal or corrected-to-normal vision. Participants received JPY 2,000 (approximately USD $17.50) as payment for their participation. They were able to win up to JPY 450 (approximately USD $3.90) based on the outcome of the prisoner’s dilemma (see below). Prior to the experiment, participants were randomly assigned to form 45 same-sex dyads without any knowledge of their assigned partners.

The participants ranged in age from 20 to 30 years, with an average age of 22 years. Of the participants, 68.9% were Japanese and 31.1% were Chinese.

### 2.2. Procedure

Upon arriving at the experiment room, each participant was greeted by an experimenter and taken to a seat. Participants were separated by a divider to ensure that they did not see each other or communicate before the conversation session began. The participants were then given a description of the study and procedures to be undertaken. After the introductory session, participants were given a consent form for review and signing. The introduction did not inform participants of the real objectives of the research or the real purpose of the experiment and instead led them to believe that they were to participate in research on personality and communication to avoid their conscious attention to their partners’ smiles and cooperation. Participants were aware that they were being videotaped but were not informed that their expressions would be coded for further analysis to avoid their conscious attention to their own smiles. Participants were not informed of the real purpose of the experiment and the plan for facial coding until after the experiment’s completion, at which point they were provided a complete description of the real aims of the research and the actual procedure of the experiment and signed a consent form to confirm that they agreed to participate in the research and to authorize the use of their personal data and video records for scientific purposes.

After signing the first consent form, participants were asked to complete two questionnaires, including a questionnaire on their current emotional state and the Altruism Scale to assess their altruism level. Then participants were told that they would participate in a one-shot prisoner’s dilemma game with another experimenter whom they had not seen, and would have no interaction with thereafter, in order to measure their cooperativeness trait with strangers. The participants believed that they would play the game with an unseen experimenter, although the results of the game were calculated between pairs and no experimenter participated in the game. Participants were assured that their decisions would be kept secret by the experimenter acting as their game partner, and that the other participants would not know their decisions. The participants were not informed of their own profits from the game until the experiment was complete.

Participants then participated in a 10-min, unstructured conversation, during which they were told that they could talk about any topic. At this time, the divider between the participants was removed and the two participants could see and talk to each other for the first time. During the conversation session, participants were seated on opposite ends of a meeting table with a 1-m distance in between them. Two Sony FDR-AX45 digital camcorders were placed approximately 0.5 m behind each participant and were used to record the facial behavior of each participant at the opposite end of the table. Participants’ facial behavior during the conversation session was video-recorded for the full 10 min at 30 frames/s with the participants’ knowledge and consent. Directly after the conversation, a visual divider was placed between the participants to separate them, and they were asked to participate in a one-shot prisoner’s dilemma game with their conversation partners and complete a questionnaire to assess their current emotional state.

### 2.3. Questionnaires

The existing literature suggests that cooperative individuals are more likely to express positive emotions than non-cooperative individuals, and that expressions of positive emotions may elicit cooperation in others (Reed et al., 2012). To examine the influence of positive emotions on smiling behavior, which, in turn, could predict cooperation, the valence, arousal, and dominance of the participants’ emotional states were measured before and after the experiment using the Self-Assessment Manikin (SAM) scale scores (Bradley and Lang, 1994). The three emotional states were rated on a 9-point scale (valence :1 = very unpleasant, 9 = very pleasant; arousal: 1 = not exciting at all, 9 = very exciting; dominance: 1 = not controlling at all, 9 = very controlling), which was illustrated using five cartoons with points listed between two figures. The participants were asked to tick the point that best corresponded to their current feelings. The difference between the pre- and post-experiment points was used as a self-reported measure of emotion.

Each participant’s general disposition toward altruism was assessed using the Self-Report Altruism Scale Distinguished by the Recipient (SRAS-DR), which was developed to evaluate altruism among Japanese university students (Oda et al., 2013). The scale contains 21 items (seven items for each recipient: family members, friends or acquaintances, and strangers) that measure the frequency at which they engage in altruistic behavior using five categories ranging from “never” to “very often.” The SRAS-DR was developed for Eastern cultures and has shown acceptable reliability and validity in both Japanese and Chinese college students. In past studies, the measure has showed high internal consistency, with a Cronbach’s alpha of 0.83 and 0.81 in Japanese and Chinese students, respectively (Oda et al., 2013; Feng and Guo, 2017). In addition, the test–retest reliability over a 1-month period was 0.83 (Oda et al., 2013). Cronbach’s alpha was 0.77 for our sample.

### 2.4. Prisoner’s dilemma game

The prisoner’s dilemma game was conducted using an exchange protocol following Shinada and Yamagishi (2014), in which, rather than choosing cooperation or defection, participants could select different levels of cooperation. The incentive structure corresponding to the prisoner’s dilemma game was presented to participants in an exchange form.

In the game, each participant was provided with an endowment of JPY 150 (approximately USD $1.31) and was asked to decide how much of the endowment to give to their game partner. The provided money was then doubled and given to the partner. Participants retained money that they did not give away. If both participants provided JPY 150 (fully cooperative), they received JPY 300. If one participant fully cooperated and provided JPY 150 and the other participant offered no money, the participant who fully cooperated earned nothing, and the one who completely defected earned JPY 450. If both participants chose to give nothing, each earned JPY 150 (mutual defection). Thus, the participant earned more by giving less, regardless of the partner’s offer level. These outcomes correspond to the four cells in the standard prisoner’s dilemma matrix. The possible outcomes (see Table 1) were clearly outlined for participants in a chart.

**TABLE 1:** The incentive structure of the prisoner’s dilemma game used in the current study expressed as a payoff matrix.

Participant A’s cooperation level (i.e., how much A gives)					
		150	100	…	0
Participant B’s cooperation level	150	300R300	200R350		0R450
	100	350R200	250R250		50R350
	…				
	0	450R0	350R50		150R150

The prisoner’s dilemma game was conducted twice: first with an assumed stranger (an experimenter who they would not interact with and they would not know each other’s information), and then with their partner after the “getting to know you” conversation. The proportion of money each participant offered before the conversation was used as a measure of altruistic cooperativeness. The proportion of money that each participant offered after the conversation was used as a measure of the individual’s cooperative behavior with the partner.

### 2.5. Behavior analysis

Analyses of facial behavior during the conversation were only conducted for 5 min directly before the prisoner’s dilemma game. By focusing on the 5 min of the clip directly prior to the prisoner’s dilemma game, we aimed to capture facial actions that were highly relevant to cooperative behavior. Ekman et al. (2002) Facial Action Coding System (FACS) was used to measure facial behavior.

Individual participants’ smiling during the 5-min clip of conversations was coded in 1-s intervals following FACS. In each second, smiles were coded as Duchenne smile (AUs 6+12, raised lip corner, presence of cheek movement, and “crow’s feet” wrinkles indicating contraction of the orbicularis oculi muscles) or non-Duchenne smile (AU 12, raised lip corner). Smiles were coded as either present or absent for each second of the 5-min clip. If a Duchenne or non-Duchenne smile was present for a second, the smile was coded as 1. Smile synchrony is defined as a series of smiles displayed simultaneously by two individuals in a dyad. At each second, smile synchrony was coded 1 if two individual smiles were presented. The results were generated as a series of binary coding for Duchenne and non-Duchenne smiles per participant separately and for smile synchrony. The indices of Duchenne smile synchrony, non-Duchenne smile synchrony, and total smile synchrony were then separately calculated as the proportion of time with Code 1 for a total of 300 s.

All videos of the 5-mn clip were coded by a certified coder. Approximately 20% of the overlapping videos were coded by another certified coder to assess reliability. The average pairwise reliability across coders, based on the intraclass correlation coefficient (ICC), was 0.917 using random effects.

## 3. Results

### 3.1. Preliminary analysis

We first examined the level of smile and cooperative behavior at the individual level and Duchenne smile synchrony at the dyadic level. The smile index was calculated as the proportion of time spent with Code 1 out of a total of 300 s. An individual’s cooperativeness was measured as the proportion of money each participant offered to an experimenter. The display of different types of smiles was significantly different [*t*(89) = 3.45, *p* < 0.001, 95% CI (−0.10, −0.02)]; generally speaking, individuals displayed significantly more non-Duchenne smiles (*M* = 0.225, SD = 0.136) than Duchenne smiles (*M* = 0.159, SD = 0.135). The average level of Duchenne smile synchrony was 0.074 (SD = 0.089). Cooperative behavior was measured as the proportion of money that each participant offered his/her partner and the sum of the proportion of money that each participant offered his/her partner at the individual and dyadic level, respectively. On average, before the conversation, participants provided the experimenter with 56.9% of the endowment of JPY 150 (*M* = 85.455, SD = 50.912), and after the conversation, participants provided their partners with 74.6% of the endowment of JPY 150 (*M* = 112.022, SD = 46.084). Participants cooperated significantly more with their partners than before the conversation [*t*(89) = 5.56, *p* < 0.001, 95% CI (−0.24, −0.11)]. Gender differences were not observed in either smiles or cooperative behavior results. Table 2 presents descriptive statistics for the variables.

**TABLE 2:** Descriptive statistics for variables.

	N	*M*	SD
Duchenne smile	90	0.159	0.135
Non-Duchenne smile	90	0.225	0.136
Duchenne smile synchrony	45	0.074	0.089
Altruistic cooperativeness	90	0.569	0.339
Post-conversation cooperative behavior	90	0.746	0.307

### 3.2. Duchenne smile and cooperative behavior

We first conducted linear regression analyses at the individual and dyadic levels to examine whether Duchenne smiles/Duchenne smile synchrony significantly predicted post-conversation cooperative behavior. Consistent with prior studies, our results showed a significant positive correlation between individual Duchenne smiles and cooperative behavior [*t*(88) = 2.29, *p* = 0.02, 95% CI (10.86, 150.64)], and a marginally significant positive correlation between synchronized Duchenne smiles and cooperative behavior [*t*(43) = 1.92, *p* = 0.06, 95% CI (−12.12, 511.61)].

We followed the four-step approach (see Table 3) proposed by Baron and Kenny (1986) that employs separate and parallel cross-sectional regression analyses to test whether Duchenne smile/Duchenne smile synchrony still significantly predicts post-conversation cooperative behavior after controlling for altruistic cooperativeness. The results showed that altruistic cooperativeness significantly predicted Duchenne smiles (*z* = 2.562, *p* = 0.01) and Duchenne smile synchrony (*z* = 3.071, *p* = 0.002). However, neither Duchenne smiles (*z* = 1.077, *p* = 0.281) nor Duchenne smile synchrony (*z* = 0.181, *p* = 0.856) significantly predicted post-conversation cooperative behavior after controlling for altruistic cooperativeness.

**TABLE 3:** Four-step approach.

Step 1	Conduct a simple regression analysis with altruistic cooperativeness (X) predicting post-conversation cooperative behavior (Y), *Y* = *B*0 + *B*1*X* + *e*
Step 2	Conduct a simple regression analysis with altruistic cooperativeness (X) predicting Duchenne smiles/Duchenne smile synchrony (*M*), *M* = *B*0 + *B*1*X* + *e*
Step 3	Conduct a simple regression analysis with Duchenne smiles/Duchenne smile synchrony (*M*) predicting post-conversation cooperative behavior (Y), *Y* = *B*0 + *B*1*M* + *e*
Step 4	Conduct a multiple regression analysis with altruistic cooperativeness (X) and Duchenne smiles/Duchenne smile synchrony (*M*) predicting post-conversation cooperative behavior (Y), *Y* = *B*0 + *B*1*X* + *B*2*M* + *e*

### 3.3. Estimating mediating effect of Duchenne smiles on cooperative behavior

**FIGURE 1:**
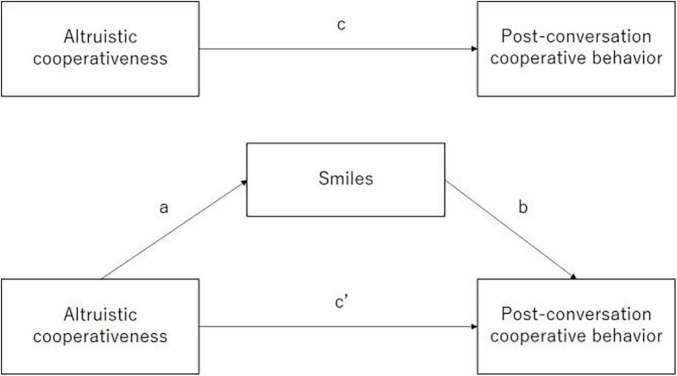
Diagram of hypothesized mediation model.

Mediation analysis was conducted following Preacher and Hayes (2004) using a bootstrapping method to determine whether the effect of the independent variable (smiler’s altruistic cooperativeness) on the dependent variable (smiler’s post-conversation cooperative behavior) can be explained by the mediating variable (smiler’s smiles toward partner). A pathway (see Figure 1) is specified *a priori*, showing the mediation model in which Path A determines the total effect of altruistic cooperativeness (independent variable) on post-conversation cooperative behavior with no consideration of mediator variables; Paths A and B determine the indirect effect of altruistic cooperativeness on post-conversation cooperative behavior through smiles and Path C determines the direct effect of altruistic cooperativeness on post-conversation cooperative behavior after removing the contribution of smiles. Mediation analysis was conducted using the mediation package in R software (version 4.0.4), which computed the total effect of the independent variable on the outcome, the average causal mediation effects (ACME) for indirect effects, and the average direct effects (ADE) for direct effects. A mediator was considered to have a mediational effect when (1) the indirect effect (i.e., Path A * Path B) of altruistic cooperativeness on post-conversation cooperative behavior via smiles was significant and (2) the bias-corrected 95% CI around the indirect effect from 5,000 bootstrap re-samples excluded zero. In the current study, two mediation analyses were conducted to test the mediating effect of (1) individual Duchenne smiles on the relationship between individuals’ altruistic cooperativeness and post-conversation cooperative behavior and (2) Duchenne smile synchrony on the relationship between total altruistic cooperativeness and post-conversation cooperative behavior of the pairs.

Table 4 presents the results of the mediation analyses at the individual and dyadic levels, which show that the total effect was significant for the association between both (1) the individual’s altruistic cooperativeness and post-conversation cooperative behavior and (2) the total altruistic cooperativeness (total effect = 0.512, *p* < 0.001) and post-conversation cooperative behavior of the pairs (total effect = 0.661, *p* < 0.001). The direct effect between altruistic cooperativeness and post-conversation cooperative behavior was significant at both the individual (ADE = 0.490, *p* < 0.001) and dyadic levels (ADE = 0.652, *p* < 0.001). However, the indirect effect of altruistic cooperativeness on post-conversation cooperative behavior via smiles was not significant at either the individual (ACME = 0.022, 95% CI: LLCI = −0.019 to ULCI = 0.08) or the dyadic level (ACME = 0.009, 95% CI: LLCI = −0.115 to ULCI = 0.14). These results suggest that either an individual’s Duchenne smile or a pair’s Duchenne smile synchrony can hardly be considered a mediator of altruistic cooperativeness post-conversation cooperative behavior.

**TABLE 4:** Mediating effect of Duchenne smile and Duchenne smile synchrony in the relationship between altruistic cooperativeness and post-conversation cooperative behavior.

	Effect	95% CI Lower	95% CI Upper	*P*-value
**Individual level (mediation effect of individual’s Duchenne smile)**				
Total effect	0.512	0.357	0.67	−2e-16***
Direct effect	0.490	0.332	0.65	−2e-16***
Indirect effect	0.022	−0.019	0.08	0.3
Path A	0.001	0.000	0.001	0.013*
Path B	32.673	−28.641	93.986	0.292
**Dyadic level (mediation effect of pairs’ Duchenne smile synchrony)**				
Total effect	0.661	0.411	0.91	−2e-16***
Direct effect	0.652	0.387	0.92	−2e-16***
Indirect effect	0.009	−0.115	0.14	0.86
Path A	0.000	0.000	0.001	0.004**
Path B	20.49	−215.376	256.357	0.862

Significant effects are marked by asterisks. *p < 0.05; **p < 0.01; ***p < 0.001.

We also examined whether Duchenne smiles and cooperative behavior were associated with the following variables: general disposition to altruism (assessed with the Self-Report Altruism Scale), valence, and arousal and dominance of participants’ emotional state changes [measured using the Self-Assessment Manikin (SAM) scale]. We did not observe any significant correlation between Duchenne smiles and self-reported altruism, valence, arousal, or dominance (*p* > 0.05). Post-conversation cooperative behavior was significantly associated only with valence [*t* = 3.755, *p* < 0.001, 95% CI (5.24, 17.03)].

### 3.4. Dynamically engaged Duchenne smiling

In addition to the above mediation analyses to test our main hypothesis, an analysis of the effect of dynamically engaging Duchenne smiles on cooperative behavior was conducted following the two-step approach of Danvers and Shiota (2018), in which the actor–partner interdependence model (APIM) was used to estimate the effects of dynamically engaged Duchenne smiling during the conversation on both the signalers and receiver’s behavior in the post-conversation prisoner’s dilemma. A measure of altruistic cooperativeness was included as a covariate in our analysis model to test whether dynamically engaged Duchenne smiling affected successive cooperative behaviors after accounting for altruistic cooperativeness.

**FIGURE 2:**
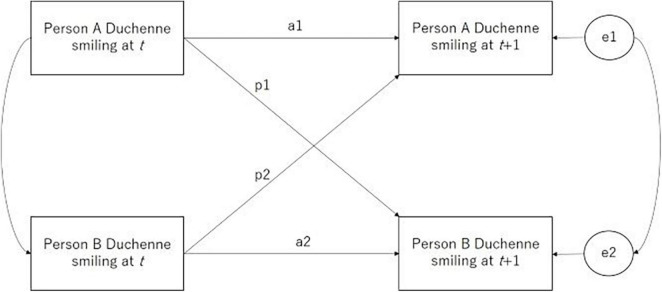
Dynamic model of Duchenne smiling engagement.

Following Danvers and Shiota (2018), cross-lagged regression models were used to predict Duchenne smiling over time for each dyad in the first step (see Figure 2), in which each person served both as an actor and a partner in the statistical analyses to maximize statistical power. Duchenne smiling for each actor at a given time point was predicted from his/her own Duchenne smiling at the previous time point and the partner’s Duchenne smiling at the previous time point. The model can estimate the extent to which a person’s Duchenne smile at time *t* influences his/her own Duchenne smile at time *t*+1 (the actor effect, denoted as a1 and a2) and estimate the extent to which a person’s Duchenne smile at time *t* influences his/her partner’s Duchenne smile at time *t*+1 (the partner effect, denoted as p1 and p2). Four parameters estimated from the model were saved for use in the next step: a1 (autoregressive effect for Person A, Person A’s independent Duchenne smiling); a2 (autoregressive effect for Person B, Person B’s independent Duchenne smiling); p1 (Person A’s cross-lagged effect on Person B, Person B’s engaged Duchenne smiling); and p2 (Person B’s cross-lagged effect on Person A, Person A’s engaged Duchenne smiling).

**FIGURE 3:**
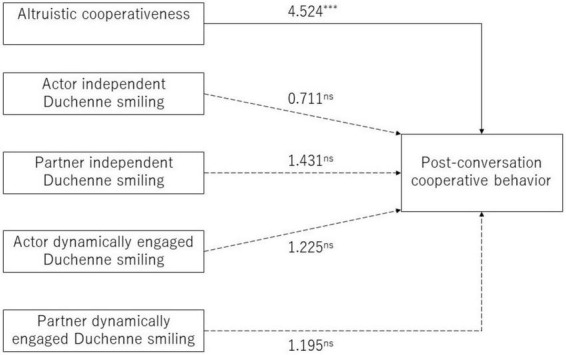
Model of the estimated effects of independent Duchenne smiling, dynamically engaged Duchenne smiling, and altruistic cooperativeness on successive cooperative behavior. Significant effects are marked by asterisks. ***p < 0.001.

In the second step, the effects of independent Duchenne smiling, dynamically engaged Duchenne smiling, and altruistic cooperativeness on successive cooperative behaviors were examined. Figure 3 illustrates the estimated structural equation model. “Actor” refers to the person whose cooperative behavior was predicted, and “partner” refers to the partner of the person whose cooperative behavior was predicted.

The results of the estimated model show that cooperative behavior is predicted only by altruistic cooperativeness. The higher the actor’s altruistic cooperativeness score, the more likely he/she was to cooperate in the post-conversation prisoner’s dilemma game (*b* = 0.370, *Z* = 4.524, *p* < 0.001). We did not observe any effect of Duchenne smiling on cooperation. Neither the degree to which the actor’s independent Duchenne smiling (*b* = 16.780, *Z* = 0.711, *p* = 0.477) nor the degree to which the actor’s Duchenne smiling was dynamically engaged with their partner (*b* = 24.372, *Z* = 1.225, *p* = 0.221) significantly predicted the actor’s own cooperative behavior. Additionally, both the degree of the partner’s independent Duchenne smiling (*b* = 35.746, *Z* = 1.431, *p* = 0.152) and the degree of the partner’s dynamically engaged Duchenne smiling with the actor (*b* = 25.764, *Z* = 1.195, *p* = 0.232) did not significantly predict the actor’s cooperative behavior.

## 4. Discussion

This study examined whether the effect of individuals’ altruistic cooperativeness traits on successive cooperative behavior can be predicted by Duchenne smiling, which is assumed to advertise altruism and elicit cooperative behavior. To this end, we measured (1) participants’ altruistic cooperativeness traits using a one-shot prisoner’s dilemma game with a stranger, (2) Duchenne smile and Duchenne smile synchrony during a 10-min, unstructured conversation, and (3) successive cooperative behavior in another one-shot prisoner’s dilemma game with the conversation partner. Our results support the hypothesis that human cooperative behavior is predicted by altruistic cooperativeness traits. Additionally, they provide support for the hypothesis that altruistic cooperativeness traits predict Duchenne smiles and Duchenne smile synchrony is predicted by altruistic cooperativeness trait. However, to our surprise, neither the Duchenne smile nor Duchenne smile synchrony predicted successive cooperative behaviors, which suggests that Duchenne smiles and Duchenne smile synchrony do not mediate the relationship between altruistic cooperativeness and cooperative behavior, and that human cooperative behavior might be predetermined by altruistic cooperativeness.

The results of the regression analyses indicated that altruistic cooperativeness significantly predicted Duchenne smiles and smile synchrony. In line with prior evidence, the association between Duchenne smiling and altruistic cooperativeness suggests that individuals who are more altruistically cooperative are more likely to display Duchenne smiling during communication, and that individual altruism might be advertised by smiling (Mehu et al., 2007a; Reed et al., 2012). Previous studies have suggested that successfully detecting potential altruistic partners through expression is key to human cooperation. Based on the reciprocal altruism theory, Duchenne smiling, as a reliable signal of altruism, should be positively associated with successive cooperative behavior for individuals to detect a cooperative partner. Consistent with prior studies, the results of our linear regression analyses showed a significant positive correlation between individual Duchenne smiles and cooperative behavior and a marginally significant positive correlation between synchronized Duchenne smiles and cooperative behavior. However, when tested after controlling for altruistic cooperativeness, neither Duchenne smiles nor Duchenne smile synchrony significantly predicted post-conversation cooperative behavior. To further examine whether Duchenne smiles work as the underlying behavioral mechanism responsible for detecting cooperators and cheaters in order to maintain the functioning of the cooperative strategy based on reciprocal altruism theory, we performed mediation models to test whether altruistic cooperativeness affects successive cooperative behavior directly or indirectly (mediated by Duchenne smiles). Surprisingly, our results showed that neither individual Duchenne smiles nor Duchenne smile synchrony significantly predicted post-conversation cooperative behavior, whereas altruistic cooperativeness significantly predicted post-conversation cooperative behavior, even when controlling for individual Duchenne smiles or Duchenne smile synchrony. Considering that Duchenne smiling might serve as a signal in the form of dynamic engagement in interaction, we also conducted an analysis using the actor–partner interdependence model (APIM) to estimate the effects of dynamically engaged Duchenne smiling on both the signaler’s and receiver’s cooperative behavior, with altruistic cooperativeness included as a covariate. Our results showed that after accounting for altruistic cooperativeness, dynamically engaged Duchenne smiling no longer affected successive cooperative behaviors. While Duchenne smiles are predicted by altruistic cooperativeness traits, it does not predict cooperative behavior, thus suggesting the possibility that Duchenne smiles might advertise altruism without being detected as a cooperative signal. In other words, our results indicate that people with cooperative traits are more likely to display a Duchenne smile, but not that Duchenne smiling can elicit cooperation. Thus, these results suggest that human altruistic cooperativeness traits are predominantly responsible for cooperative behavior.

One potential explanation for these results is that Duchenne smiles are not the only emotional expressivity that the cooperator tends to display. As Schug et al. (2010) showed, cooperators display greater numbers of both positive and negative emotional expressions. The tendency of cooperators to display more Duchenne smiles, as reported in previous studies (Mehu et al., 2007a; Reed et al., 2012; Danvers and Shiota, 2018), may be because cooperators tend to openly express all their emotions in different scenes. For example, negative emotions expressed by cooperators in response to shared unfair situations may reflect prosocial preferences (Fehr et al., 2002). In the current study, participants participated in a 10-min unstructured conversation during which they discussed various topics that might not reflect only positive emotions. Future studies investigating both positive and negative emotional expressions may provide better insights into the role of facial expressions in cooperative interactions.

Another potential explanation lies in the classification of smiles. In this study, we used the classic approach developed by Ekman and Friesen (1978) to distinguish between smiles that reflected an underlying positive affect (true or genuine smiles, Duchenne smiles) and those that did not (false or fake smiles, non-Duchenne smiles). Although this approach is widely used by researchers, as classic studies have demonstrated that Duchenne smiles occur during states of happiness and non-Duchenne smiles are employed to deliberately mask negative feelings (Ekman and Friesen, 1982; Duchenne and de Boulogne, 1990), there has recently been an increasing number of studies that challenge the propriety of parsing smiles according to whether they reflect true or faked positive emotions because humans display smiles in various emotional states, such as distress, pain, pride, and embarrassment (Keltner, 1995; Ansfield, 2007; Tracy and Matsumoto, 2008; Kunz et al., 2009). In the current study, we cannot reject the possibility that smiles classified as Duchenne include both positive and negative emotional expressivity, which have different social functions across contexts. Considering our finding of a significant association between valence and cooperative behavior, further research is needed to isolate Duchenne smiles expressing positive emotions from other emotional states to test their effect on the relationship between altruism and cooperation.

Another possible explanation is that deliberate Duchenne smiles interfered with the prediction of subsequent cooperative behavior. Recently, increasing evidence suggests that some people can produce Duchenne smiles deliberately (Gunnery et al., 2013; Krumhuber et al., 2014). Therefore, those who can deliberately employ a Duchenne smile can use it strategically to cheat their partners into cooperation, even though they have no true intention to cooperate. Although people with the ability to produce deliberate Duchenne smiles are reported to be a minority, this deliberate Duchenne smile might affect the association between Duchenne smiles and cooperative behavior. Therefore, further studies are needed to test whether deliberate Duchenne smiles in a general sample affect the calculation of the relationship between Duchenne smiles and cooperative behavior.

Our results suggest that variance in the Duchenne smile and its synchrony during conversation, which are observed naturally between individuals and conversation groups in the same conversation setting, do not contribute to later cooperative behavior with the conversation partner after accounting for dispositional altruistic cooperativeness. However, this does not exclude the possibility that cooperative behavior can be enhanced by deliberately augmenting smiles and their synchrony within individuals and groups. Future studies could test this possibility by manipulating the appearance and perception of smiling in a within-subjects manner. For example, using avatar technologies in virtual space communications, a recent study reported that individuals exhibited positive emotions when their smiles were imitated by a virtual agent, regardless of their belief that the agent was a computer program (Numata et al., 2020).

It is also possible that other modes of non-verbal behavior (e.g., gaze patterns and vocal characteristics) influence how smiles are interpreted or related to cooperative decisions. This idea is partially supported by our previous and preliminary study, which reported that direct gaze during synchronized Duchenne smiling predicted the likelihood of cooperation between the signaler and receiver in the prisoner’s dilemma game (Deng et al., 2022). Future studies would benefit from considering other modes of non-verbal behavior that accompany smiles during communication when testing their effect on cooperation.

Caution is required when interpreting the current study for several reasons. First, similar to many studies on smiling and cooperation, our participants were limited to university and college students. In addition, although our participants were Chinese and Japanese, they were limited to one ethnic group, East Asian, which shares many cultural aspects. Some cross-cultural studies have suggested that people’s cultures shape how they judge smiles (Matsumoto and Kudoh, 1993; Tsai et al., 2019). Further research should be conducted on a more diverse and representative population to reach a broader generalization. Finally, we cannot exclude the possibility that the participants made their decision to cooperate or to compete based on the contents of their conversation. Further studies are needed to examine the effect of smiles on cooperative behavior without the interference of conversation.

In summary, this study investigated the predictive relationship between altruistic cooperativeness traits, successive cooperative behavior, and Duchenne smiles. The results of analysis models support the hypothesis that human cooperative behavior is predicted by individuals’ altruistic cooperativeness. However, contrary to our hypothesis, Duchenne smiles did not mediate the relationship between altruistic cooperativeness and cooperative behavior. Our results showed that while Duchenne smiles and Duchenne smile synchrony are predicted by altruistic cooperativeness traits, they do not predict successive cooperative behavior. Taken together, these results suggest that Duchenne smiles during conversations are predicted by individuals’ altruistic cooperativeness, but these results were not encouraging in interpreting Duchenne smiles simply as a signal of people’s cooperative intent. Cooperative behavior within the context of interaction may be predetermined by altruistic cooperativeness, which can be expressed in wider modes of non-verbal behavior than the subtle differences in smile expressions.

## Data availability statement

The raw data supporting the conclusions of this article will be made available by the authors, without undue reservation.

## Ethics statement

The studies involving humans were approved by the Human Subjects Research Ethics Review Committee of Tokyo Institute of Technology. The studies were conducted in accordance with the local legislation and institutional requirements. The participants provided their written informed consent to participate in this study.

## Author contributions

XD developed the study concept and design, and wrote the manuscript and analyzed the data, which was then interpreted by XD and TN. XD and SH conducted the data collection and behavioral coding. YM provided the conceptual advice on the study. TN supervised the study and experimental design and provided the advice on the concept, analytical methods, results, and overall manuscript. All authors discussed the results and commented on the manuscript.
